# Atherosclerotic plaque detected by transesophageal echocardiography is an independent predictor for all-cause mortality

**DOI:** 10.1007/s10554-020-01840-6

**Published:** 2020-04-16

**Authors:** Houtan Heidari, Hong Ran, Georg Spinka, Christian Hengstenberg, Thomas Binder, Georg Goliasch, Matthias Schneider

**Affiliations:** 1 Department of Internal Medicine II, Medical University of Vienna, Waehringer Guertel 18-20, 1090 Wien, Austria; 2 Department of Echocardiography, Nanjing First Hospital Affiliated to Nanjing Medical University, Nanjing, China

**Keywords:** Transesophageal echocardiography, Aortic plaque, Survival

## Abstract

Atherosclerotic lesions in the great arteries are frequent findings in the elderly. Numerous studies have shown their strong predictive value for cardiovascular disease, embolic events, and mortality. We sought to determine the risk of all-cause mortality depending on the localization of plaques in the thoracic aorta evaluated by transesophageal echocardiography (TEE). A total of 2,054 patients (median age 65 years, interquartile range 52–73; 58% men) who underwent a TEE examination between 01/2007 and 03/2015 were retrospectively analyzed. For each patient, the presence of atherosclerotic lesions in the ascending aorta, the aortic arch, and in the descending aorta, as well as cardiovascular risk factors and survival were documented. Median follow-up period was 48 months (interquartile range 38–58). Multivariate Cox regression analysis indicated plaque in the ascending aorta (HR of 1.36, 95% CI 1.01–1.83, P = 0.046), the aortic arch (HR of 1.78, 95% CI 1.29–2.45, P < 0.001), the descending aorta (HR of 2.01, 95% CI 1.54–2.77, P < 0.001), and plaque in any part of the thoracic aorta (HR of 1.84, 95% CI 1.42–2.4, P < 0.001), as independent predictors for all-cause mortality after adjusting for age, sex, arterial hypertension, hyperlipidemia, smoking, and diabetes. In this study, we could demonstrate that more than mild plaque at any site of the thoracic aorta predicts all-cause mortality. Assessment of atherosclerotic lesions in all segments of the thoracic aorta should be part of every routine TEE examination.

**Electronic supplementary material:**

The online version of this article (10.1007/s10554-020-01840-6) contains supplementary material, which is available to authorized users.

## Introduction

Atherosclerosis and its cardiovascular sequelae represent the leading cause of death globally.

A common manifestation of systemic atherosclerosis is atheroma of the thoracic aorta, a well-acknowledged cause of embolic events [1–3]. Numerous studies have shown the strong correlation of severe thoracic aortic plaque and cerebrovascular events [4], especially in patients with cryptogenic or recurrent stroke [5]. Complex and large (> 4 mm) plaques have a particularly high risk for embolism [6]. Furthermore, aortic atheroma is associated with the presence of aortic stenosis [7], atrial fibrillation [8], mitral annular calcification [9], carotid artery disease [10], and coronary artery disease [11]. One small study showed that plaque in the descending aorta predicts cardiovascular events [12], another showed the prognostic value of large plaque lesions in the aortic arch [13].

It is well recognized that plaque may occur at any level of the thoracic aorta and its prevalence increases with age [14]. However, the Stroke Prevention: Assessment of Risk in a Community (SPARC) study identified the descending aorta as the most prevalent site of aortic atherosclerosis (34.4%) followed by the aortic arch (25.6%). The ascending aorta was only affected in 6.2% of included patients [15]. So far, no studies have explored the plaque extent in different levels of the thoracic aorta with long-term survival.

Although there has been evidence for plaque in the thoracic aorta as a risk factor for long-term survival, it is poorly understood whether even small plaque detected in TEE predicts mortality in an unselected population.

We sought to investigate the prognostic value of plaque assessed with TEE depending on its localization in the thoracic aorta.

## Methods

### Study design, patient selection, and data acquisition

**Fig. 1:**
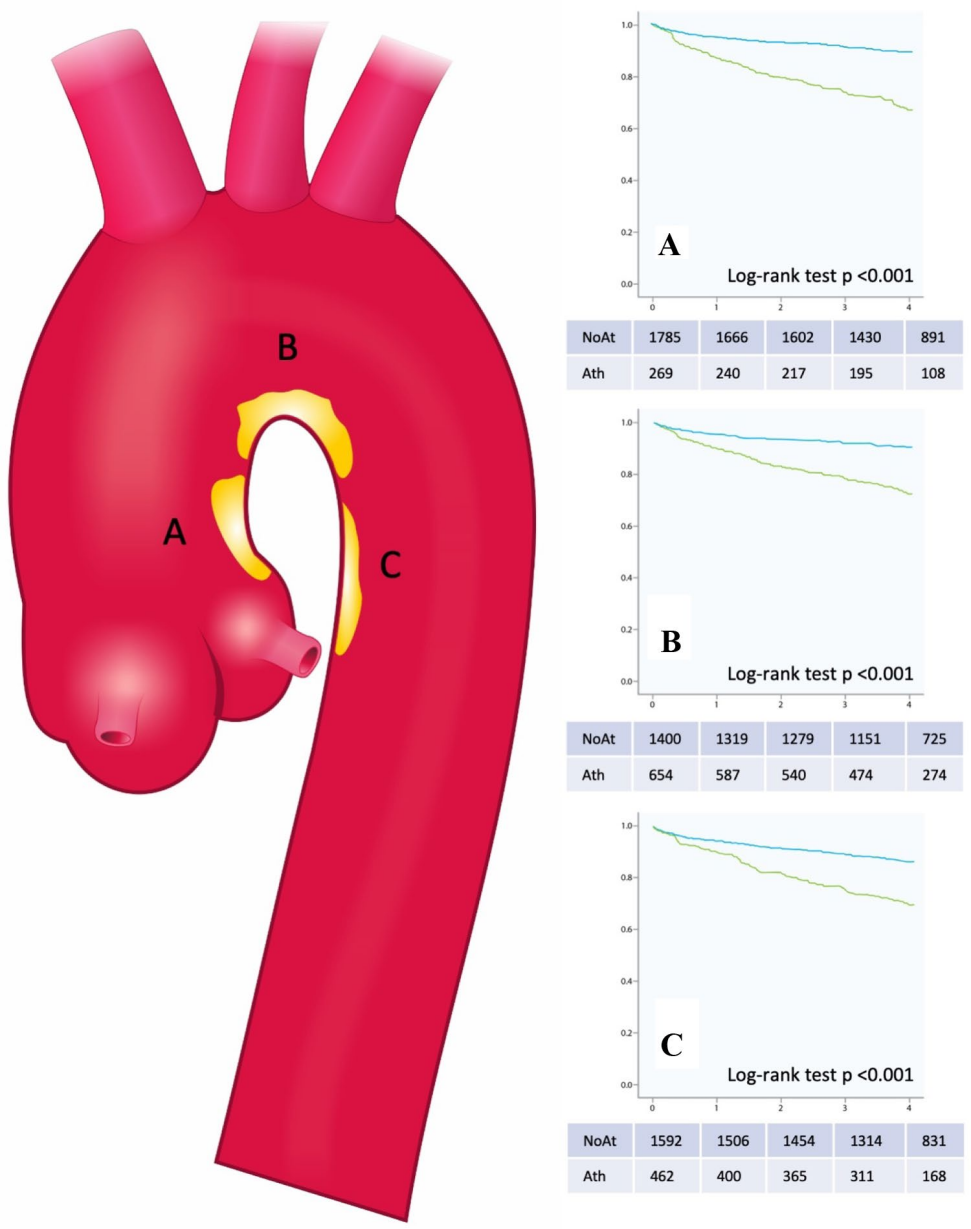
Kaplan Meier curves as well as tables with patients at risk showing 4-year-survival of patients with and without plaque in the different regions of the thoracic aorta. **a** Ascending aorta. **b** Aortic arch. **c** Descending aorta. *NoAt *no atheromatosis, *Ath *atheromatosis

We conducted a retrospective single center cohort study to identify the impact of plaque in the thoracic aorta on all-cause mortality. All patients who received a TEE at our institution between 01/2007 and 03/2015 and where plaque location and extent in the thoracic aorta were clearly stated in the written report were included in the study. The primary endpoint was defined as all-cause mortality. Survival until 12/31/2017 was documented via the “Statistik Austria” death registry. Survival time was calculated from the date of the TOE examination until 12/31/2017 in all patients. Patient demographics, medical diagnoses, and daily medication were retrieved from the centralized patient management system of Vienna (AKIM- AKH-Informationsmanagement). The following clinical data were collected: age, sex, presence of arterial hypertension, dyslipidemia, diabetes mellitus, smoking, and cardiovascular medications (oral anticoagulants, antiplatelet medication, ACE inhibitors/ AT1 blocker, beta blockers, diuretics). Smoking was defined as no-smoker or as current/ former smoking habit. The study was approved by the ethics committee of the Medical University of Vienna (No. 1725/2016) and complies with the 1975 declaration of Helsinki (Fig. 1).

### Echocardiography

**Fig. 2:**
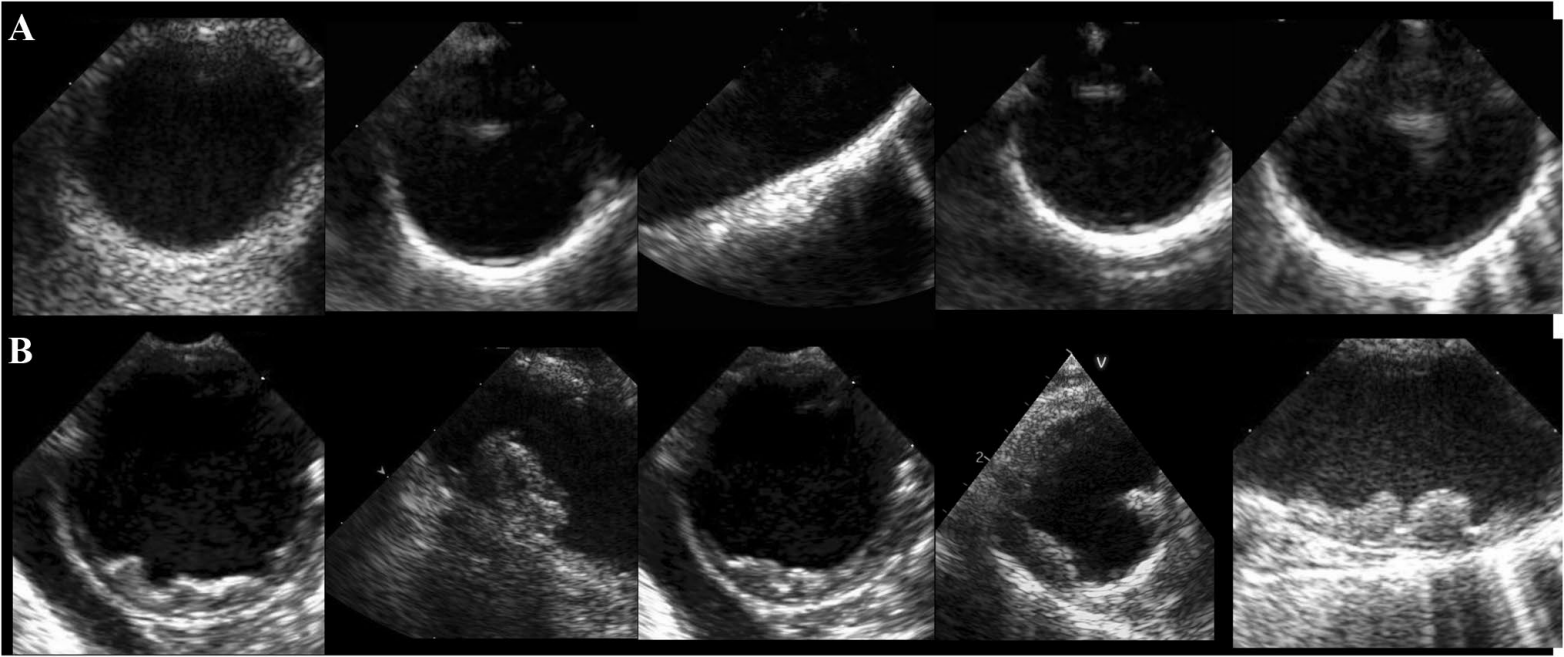
Representative image of patients with none or mild plaque (**a**) and with more than mild plaque (**b**: relevant plaque)

TEE data was retrieved from our echocardiography documentation system. All included patients underwent a complete standardized examination performed by cardiologists experienced in this modality. The plaque extend was assessed by visual estimation and reported as no plaque, mild plaque, mild to moderate plaque, moderate plaque, moderate to severe plaque, and severe plaque. Since data analysis included examinations from a time period of eight years, plaque extent was neither judged by the same readers nor was there a single definition of cut-offs regarding plaque size or plaque thickness. To minimize interobserver variability, we did not differentiate between different degrees of plaque but compared presence or absence of relevant plaque (Fig. 2). Previous studies revealed no increased mortality risk in patients with small plaque [16]. To establish the two study groups *relevant* and *non-relevant* plaque, we therefore counted no plaque and mild plaque as non-relevant, any degree of plaque more than mild was counted as relevant plaque.

The thoracic aorta was divided into three segments: the ascending aorta (proximal the brachiocephalic artery), the aortic arch (between the origin of the brachiocephalic and the left subclavian artery) and the descending aorta (distal from the left subclavian artery).

### Statistical analysis

Continuous data are given as median and standard deviation (SD). Discrete data are presented as counts and percentages. Multivariate Cox proportional hazards regression analysis was applied to assess the association between atheroma in the different segments of the thoracic aorta with all-cause mortality. The regression models were adjusted for the following confounders: age, sex, arterial hypertension, diabetes, hyperlipidemia, and smoking. Schoenfeld residuals was calculated to check the proportional hazards assumption. Kaplan Meier Curves are presented for survival analysis. Unpaired student’s t-test was applied to compare categorical data. A P value of < 0.05 was considered statistically significant. Statistical analyses were performed using SPSS Version 24 (IBM SPSS, USA).

## Results

### Baseline characteristics

A total of 2054 patients were included in the study, 58% were male. Median age was 65 years (interquartile range 52–73). All patients underwent a complete TEE study. Cardiovascular risk factors were significantly present with 62% suffering from arterial hypertension, 41% had hyperlipidemia, 15% suffered from diabetes mellitus, and 31% were former or active smokers. Cardiovascular risk factors as well as cardiovascular medication were significantly more frequently present in patients with than in those without atheromatosis. Detailed baseline characteristics of all patients are displayed in Table 1.

**Table 1:** Patient characteristics

Patient characteristics	Total	Mild to no atheromatosis of the aorta	> Mild atheromatosis of the aorta	P value
Number of patients, n (%)	2054	1190 (58)	864 (42)	
Age, median years (Q1–Q3)	65 (52–73)	57 (46–67)	72 (65–77)	< 0.001
Male sex, n (%)	1199 (58)	680 (57)	519 (60)	0.155
Cardiovascular risk factors				
Arterial hypertension, n (%)	1281 (62)	609 (51)	672 (78)	< 0.001
Diabetes mellitus, n (%)	301 (15)	108 (9)	193 (22)	< 0.001
Hyperlipidemia, n (%)	838 (41)	413 (35)	425 (49)	< 0.001
Smoking, n (%)	633 (31)	321 (27)	312 (36)	< 0.001
Medication				
Anticoagulation, n (%)	787 (38)	422 (36)	365 (42)	0.003
Antiplatelet therapy, n (%)	820 (40)	400 (34)	420 (49)	< 0.001
ACE inhibitor, n (%)	1024 (50)	480 (40)	544 (63)	< 0.001
Beta blocker, n (%)	1024 (50)	502 (42)	522 (60)	< 0.001
Diuretics, n (%)	789 (38)	330 (28)	459 (53)	< 0.001

*ACE* angiotensin-converting-enzyme

### Echocardiographic findings

The thoracic aorta was assessed in all patients. Of the 2054 included patients, 42% had more than mild plaque at any level of the thoracic aorta, 13% had plaque in the ascending aorta, 32% in the aortic arch, and 23% in the descending aorta. The group of patients which did not survive 4-year follow-up had significantly higher numbers of atheromatosis than the group that survived (Table 2).

**Table 2:** Patient characteristics and echocardiographic data

Echocardiographic data	Total	Survived 4 year FU	Did not survive 4 year FU	P value
Number of patients, n (%)	2054	1711 (83)	343 (17)	
Plaque thoracic aorta, n (%)	864 (42)	634 (37)	230 (67)	0.002
Plaque AscAo, n (%)	268 (13)	189 (11)	79 (23)	< 0.001
Plaque AoArch, n (%)	654 (32)	485 (28)	169 (49)	< 0.001
Plaque DesAo, n (%)	462 (23)	318 (19)	144 (42)	< 0.001
Aortic stenosis ≥ mild, n (%)	342 (17)	225 (13)	117 (34)	< 0.001
MAC, n (%)	188 (9)	142 (8)	46 (13)	< 0.001

*FU* follow-up, *AscAo* ascending aorta, *DesAo* descending aorta, *AoArch* aortic arch, *MAC* mitral annular calcification

### Prediction of long-term mortality

Over a median follow-up period of four (SD ± 2,2) years, 17% of patients died. Kaplan–Meier curves for all-cause mortality are shown in Fig. 1.

In a multivariate cox regression analysis adjusting for age, sex, arterial hypertension, hyperlipidemia, smoking, and diabetes, plaque in the thoracic aorta remained independently associated with a dismal outcome regardless of the plaque location (Table 3).

**Table 3:** Prognostic value of plaque in the different levels of the Aorta: Multivariate analysis, adjusting for age, sex, cardiovascular risk factors (diabetes, hypertension, hyperlipidemia, smoking), and presence of aortic plaque at different levels as predictors for survival

Variable	HR	95% CI	P value
Plaque in the AscAo	1.36	1.01–1.83	**0.046**
Plaque in the AoArch	1.78	1.29–2.45	**< 0.001**
Plaque in the DescAo	2.01	1.54–2.77	**< 0.001**
Plaque in any part of the thoracic aorta	1.84	1.42–2.4	**< 0.001**
Plaque in all parts of the thoracic aorta	0.83	0.76–1.25	0.829

*AscAo* ascending aorta, *AoArch* aortic arch, *DescAo* descending aorta, *HR* hazard ratio, *CI* confidence interval

## Discussion

The present study demonstrates that more than mild plaque in any of the segments of the thoracic aorta is independently associated with a dismal outcome.

### Prognostic value of plaque

Plaque in the thoracic aorta is a common finding in the routinely performed transesophageal echocardiogram, especially in the elderly [17]. An association with cardiovascular risk factors has been demonstrated previously [18]. In the present study, we showed an association with hypertension, diabetes mellitus, dyslipidemia, and smoking.

Most previous studies used severe plaque (> 4/5 mm) as the cut-off and did not take smaller plaque into account [2, 19]. Limited data are available regarding the association of smaller plaque in the thoracic aorta detected by TEE with all-cause mortality. Previous studies were mostly limited to cardiac surgery cohorts [8, 20], patients with coexisting atrial fibrillation [13], or used other modalities such as computed tomography [20] or transthoracic echocardiography [21, 22]. Ferrari et al. demonstrated in a prospective study including 129 patients, that even small plaques (1–3.9 mm) are associated with embolic events and mortality in an unselected population [21]. With our data, we can confirm these findings in a large cohort. Multivariate analysis demonstrated significant prognostic power for all three segments of the thoracic aorta independent of age, sex, and cardiovascular risk factors. Kaplan–Meier curves revealed a significantly elevated mortality risk for patients with plaque in any region of the thoracic aorta.

### Different plaque regions

The distribution of plaque along the thoracic aorta in our cohort was similar to previously published reports, which described the arch and the descending aorta as the more frequently affected sites when compared to the ascending aorta [15]. One hypothesis to explain this phenomenon is the differing wall sheer stress along the thoracic aorta [22].

An observational study including more than 22,000 patients demonstrated that plaque of any size in the descending and ascending aorta is a significant risk factor for long-term survival in cardiac surgery patients [23]. However, to the best of our knowledge, ours is the first study to analyze the impact of plaque in the three segments of the thoracic aorta on all-cause mortality in an unselected large population. There are several reports describing the impact of plaque in the different locations of the aorta on cardiovascular diseases. Atheroma in the ascending aorta and the aortic arch are considered main risk factors for stroke [5]. Patients with aortic arch atheroma were shown to have a similar stroke prevalence as patients with atrial fibrillation and carotid stenosis [24]. Furthermore, plaque in the proximal thoracic aorta is considered a major risk factor for recurrent stroke [5] and embolic events related to cardiac procedures (TAVI, cardiac surgery) [23, 25]. Plaque in the descending aorta can also be an indicator for systemic atherosclerotic disease. This assumption is supported by the fact that there is a strong association with age and cardiovascular risk factors [26], and with coronary artery disease [11]. Further investigations are needed to clarify a possible causal link of plaque in the descending aorta with embolic events and mortality.

The knowledge of plaque extent and distribution has several clinical implications. Aortic atheroma is strongly associated with cardiovascular risk factors. Therefore, secondary prevention with optimal treatment of arterial hypertension, diabetes mellitus, and dyslipidemia as well as smoking cessation are of even more importance in this patient group. The knowledge about plaque burden may furthermore provide information to anticipate the patient’s risk for embolic events and mortality during cardiac procedures. Finally, Ferrari et al. were able to demonstrate the superiority of anticoagulation versus antiplatelet therapy in patients with severe aortic plaque (> 4 mm). However, they did not show a beneficial effect in the group with plaque size of 1–3.9 mm [21]. Further studies are necessary to clarify the optimal management of these patients.

### Limitations

The present study has several limitations. The data collection was retrospective; the reported values were exported from the hospital’s database and not confirmed by an independent echocardiographer. Our data reflects the experience of a single tertiary care center. However, the potential advantage of a single-center approach is the consistent quality of imaging. We included all consecutive patients undergoing TEE into our study regardless of age, indication, and medical history. However, a cohort with an indication for TEE is not comparable to the general population. Plaque extend in our cohort was assessed by visual estimation by different cardiologists. To minimize this potential limitation, we did not differentiate between different degrees of plaque but compared presence or absence of relevant plaque.

The assessment of the aorta via transesophageal echocardiography is limited by the “blind spot”, which refers to a small area of the distal ascending aorta which cannot be seen due to the interposition of the air-filled trachea between the esophagus and the aorta [27, 28]. In addition, especially in the distal ascending aorta, the aortic arch, and in the proximal descending aorta, due to the proximity of the probe to the aorta, there can be a near field drop out artefact of the aortic wall close to the probe. Even though there are some regions which cannot or can only partly be evaluated, nevertheless the most of the three parts of the aorta are accessible in most patients and give a representative impression on the degree of overall calcification.

### Conclusion

In this study, we could demonstrate that more than mild plaque at any site of the thoracic aorta predicts all-cause mortality. Assessment of atherosclerotic lesions in all segments of the thoracic aorta should be part of every routine TEE examination.

## Electronic supplementary material

Below is the link to the electronic supplementary material.

Supplementary file1 (MP4 3519 kb)

Supplementary file2 (MP4 3475 kb)

Supplementary file3 (MP4 3011 kb)
